# Optimization of Base-Catalyzed Transesterification toward Maximum Biodiesel Yield from *Camelus dromedarius* Fat Waste

**DOI:** 10.1021/acsomega.6c03814

**Published:** 2026-10-29

**Authors:** Sami Alsaadi, Mardiana Idayu Ahmad, Fozy Binhweel, Wardah Senusi, Ehsan Shalfoh, Mohammad Aliff Shakir

**Affiliations:** † Environmental Technology Division, School of Industrial Technology, 26689 Universiti Sains Malaysia, Gelugor, Penang 11800, Malaysia; ‡ Biology Department, College of Science, University of Hail, Hail 81422, Saudi Arabia; § Renewable Biomass Transformation Cluster, School of Industrial Technology, Universiti Sains Malaysia, Gelugor, Penang 11800, Malaysia; ∥ Faculty of Environmental Science and Marine Biology, Hadhramout University, Mukalla, Hahdramout 50512, Yemen

## Abstract

Utilization of organic waste offers a promising pathway to advance the circular bioeconomy and address global energy challenges through sustainable biodiesel production. *Camelus dromedarius* fat waste, abundant in arid and semiarid regions, remains an underexploited byproduct with considerable potential as a renewable biodiesel feedstock, reducing dependence on conventional resources. Despite significant progress in biodiesel research, studies on the transesterification of *Camelus dromedarius* fat waste are limited, and comprehensive optimization of its reaction parameters is lacking. This study evaluated biodiesel production from camel fat waste oil through alkali-catalyzed transesterification and optimized the process using Response Surface Methodology (RSM) based on a Central Composite Design (CCD). For the optimization stage, catalyst concentration (0.25–1.25 wt %), reaction temperature (45–85 °C), reaction time (15–55 min), and oil-to-methanol molar ratio (1:3–1:15) were selected as independent variables to investigate their individual and interactive effects on biodiesel yield. The RSM model predicted a maximum biodiesel yield of 95% under optimal conditions of 68 °C reaction temperature, 0.8 wt % catalyst concentration, 36 min reaction time, and an oil-to-methanol molar ratio of 1:9.6. Validation experiments confirmed the model prediction with an average yield of 94.82% and a relative error of only 0.19%. The produced biodiesel was characterized by FTIR, GC-FID, and physicochemical properties. The fuel properties were largely consistent with ASTM D6751 specifications, while viscosity slightly exceeded the EN 14214 limit and cold-flow properties indicated limited suitability for low-temperature applications. This underscores the potential of discarded camel fat as a sustainable feedstock and demonstrating that optimized transesterification enhances resource efficiency and advances biodiesel technologies within a circular bioeconomy framework.

## Graphical abstract

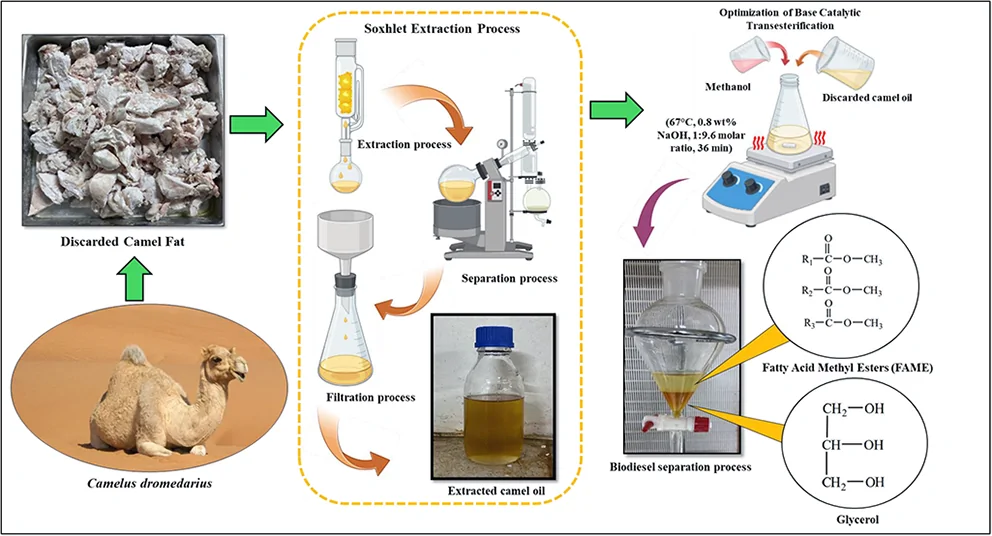

## 1 Introduction

Rising global energy demand and concerns about greenhouse gas emissions have intensified the search for renewable and sustainable alternatives to petroleum-derived fuels. Biodiesel has emerged as one of the most promising renewable fuels because it can be produced from renewable lipid sources and used directly or blended with conventional diesel, reducing dependence on fossil fuels and lowering environmental impacts.^1^ Global biodiesel production reached approximately 63 billion liters in 2023, demonstrating its increasing contribution to the renewable energy sector.^2^

The commercial viability of biodiesel as a petroleum diesel substitute largely depends on the availability and cost of feedstock, which account for approximately 70–95% of total production expenses. On the basis of feedstock origin and technological development, biodiesel feedstocks are commonly classified into four generations, although this classification is not universally standardized.^3^ First-generation feedstocks include edible oils, although their use raises sustainability concerns due to competition with food resources and environmental impacts. Despite these concerns, edible oils still account for approximately 95% of the global biodiesel production. Second-generation feedstocks, such as jatropha and mahua, offer better sustainability but are limited by seasonal and geographic factors.^4^ Third-generation feedstocks including algae-derived oils, waste cooking oil, and animal fat wastes are particularly attractive because of their abundance, economic feasibility, and contribution to waste valorization.^5^ Fourth-generation feedstocks are based on genetically engineered microorganisms and advanced biotechnological approaches designed to enhance lipid productivity and sustainability.^6^ However, these technologies remain at an early stage of development, and their commercial implementation is still limited.^7^ It should be noted that alternative classification schemes have also been proposed in the literature, and therefore, the four-generation framework should be regarded as one of the commonly adopted approaches rather than a universally accepted classification.^8^

Animal fats are an abundant and underutilized feedstock for biodiesel production, offering a sustainable and cost-effective alternative to conventional vegetable oils.^9,10^ Several studies have demonstrated the feasibility of converting animal fat wastes into biodiesel, including beef tallow,^11^ fish waste,^12^ chicken waste fat,^13^ goat fat waste,^14^ and sheep fat waste.^15^ Collectively, these studies underscore the potential of animal fat wastes as sustainable feedstocks that contribute to both effective waste management and renewable energy production.^16^ *Camelus dromedarius* fat waste represents an abundant yet underutilized animal byproduct, particularly in Middle Eastern and North African countries where camel meat consumption is widespread. Large quantities of camel fat are generated by slaughterhouses and food-processing industries, and improper disposal may contribute to environmental pollution. Converting this waste into biodiesel offers a sustainable waste management strategy while producing a renewable fuel. Despite this promising evidence, research on *C. dromedarius* fat waste remains limited, highlighting a clear feedstock knowledge gap. Sbihi et al.^17,18^ investigated this feedstock for oil characterization and biodiesel synthesis, demonstrating its viability as a promising biodiesel source.

Optimizing the transesterification process is vital for achieving a high biodiesel yield and improving process efficiency. Base-catalyzed transesterification is widely used due to its high yield, and statistical tools such as response surface methodology (RSM) enable systematic optimization of reaction parameters while minimizing cost and resource use.^19–21^ Efficient transesterification is critical, as it accounts for the majority of biodiesel production success.^22,23^ Several studies have applied optimization techniques to biodiesel production from various animal fat wastes, underscoring the importance of statistical and process modeling approaches in enhancing the yield and fuel quality. Mohamed et al.^24^ reported biodiesel yields exceeding 89.07% from waste fish oil using RSM, while Binhweel et al.^4^ achieved a maximum yield of 95.15% from discarded beef tallow through base-catalyzed transesterification. Similarly, an RSM-based study on chicken fat biodiesel attained a 95% yield under optimized conditions.^25^ Yet, comparable studies of *Camelus dromedarius* fat waste remain scarce. Addressing this optimization gap is therefore essential to enhancing biodiesel yield, cost-effectiveness, and sustainability, supporting the valorization of camel fat waste as a viable feedstock for renewable energy production.

In this context, the present study addresses the existing research gap by providing a comprehensive optimization of biodiesel production from *Camelus dromedarius* fat waste using a central composite design (CCD)-based response surface methodology (RSM). The contribution of this study centers on *C. dromedarius* fat waste as an underutilized animal-derived feedstock, for which the lipid characteristics and corresponding transesterification requirements differ from those of previously investigated feedstocks. Accordingly, the present work provides a feedstock-specific assessment of how the fatty acid composition of *C. dromedarius* fat waste relates to its transesterification behavior and the physicochemical properties of the resulting biodiesel. Unlike previous studies that primarily demonstrated the feasibility of biodiesel production from camel fat, this work systematically investigates the combined effects of the methanol-to-oil ratio, catalyst concentration, reaction temperature, and reaction time to establish statistically validated optimum operating conditions. The extracted oil and synthesized biodiesel were comprehensively characterized in terms of fatty acid composition, chemical functionality, and fuel properties, and their compliance with ASTM D6751 and EN 14214 biodiesel specifications was evaluated. Finally, the performance of the produced biodiesel was compared with that of biodiesels derived from other waste animal fat feedstocks to further assess the potential of *Camelus dromedarius* fat waste as a sustainable and renewable biodiesel resource.

## 2 Materials and Methods

### 2.1 Sample Preparation and Oil Extraction

The *Camelus dromedarius* fat waste samples were collected from an Arab restaurant in Johor Bahru, Malaysia. The fat was thoroughly washed and cut into 1–3 cm^3^ pieces for dehydration, which was performed by freeze-drying at −50 °C for 72 h. Freeze-drying was selected over other techniques due to its superior ability to reduce the water content of the fat sample from 19.51% to 2.18%. The drying duration of 72 h was established through preliminary experiments, which showed that extending the drying time beyond this period did not produce any further measurable decrease in moisture content, indicating that moisture equilibrium (constant moisture content) had been reached.^26^ After dehydration, the fat waste was crushed into particles smaller than 1 mm and stored at temperatures below −4 °C until further use.

Soxhlet extraction was employed to extract oil from *Camelus dromedarius* fat waste. Approximately 50 g of the prepared fat sample was placed into a cellulose thimble and then placed in the extraction chamber. A hexane-methanol mixture at a volume ratio 1:1 was used as the solvent. The extraction was carried out at 70 °C for 5 h under controlled conditions. After extraction, the solvent flask was placed in a rotary evaporator to separate the oil from the solvents. The obtained oil was subsequently filtered to remove impurities and any residual sample particles. The extraction percentage was calculated based on eq 1.

1 $$\mathrm{Extraction percentages}\left(\%\right)=\frac{\mathrm{Separated oil weight}}{\mathrm{Sample weight}}$$

A hexane:methanol (1:1, v/v) solvent mixture was selected because it provides efficient extraction of both polar and nonpolar lipid fractions from animal fat matrices. Hexane effectively dissolves nonpolar lipids such as triglycerides, whereas methanol disrupts lipid–protein interactions and enhances the extraction of more polar lipid constituents, thereby improving the overall lipid recovery. Compared with hexane or petroleum ether used individually, the mixed solvent system generally offers a higher extraction efficiency for complex biological materials. Although the chloroform:methanol solvent system is used widely for lipid extraction, the substitution of chloroform with hexane reduces solvent toxicity and environmental concerns while maintaining satisfactory extraction performance. Therefore, the hexane:methanol mixture was considered an appropriate compromise among extraction efficiency, operational safety, and environmental considerations.^27^

### 2.2 Transesterification

A base-catalyzed transesterification process was performed on the extracted camel oil to produce biodiesel, as illustrated in Figure 1. In this process, *Camelus dromedarius* fat waste oil reacted with methanol (CH_3_OH) using sodium hydroxide (NaOH) as the catalyst. NaOH was selected because of its high catalytic activity, low cost, commercial availability, and proven effectiveness in biodiesel production from low-free-fatty-acid feedstocks. Compared with KOH, NaOH provides comparable catalytic performance while being more economical. Heterogeneous catalysts were not considered because they generally require higher reaction temperatures, longer reaction times, and often exhibit lower catalytic activity than homogeneous alkaline catalysts. Acid catalysis was also not employed because the extracted camel fat oil had a sufficiently low free fatty acid (FFA) content, making alkaline transesterification more suitable. Although NaOH is susceptible to soap formation in the presence of high FFA and moisture, these effects were minimized by using dried oil with low FFA content. The reaction was governed by four key variables: oil-to-methanol molar ratio, catalyst concentration, reaction temperature, and reaction time. These parameters were investigated within the following ranges: 1:3–1:12 mol/mol for oil-to-methanol ratio, 0.25–1 wt % for catalyst concentration, 55–70 °C for temperature, and 15–60 min for reaction time, as summarized in Table 1. The selected ranges were established through preliminary experiments and supported by previous studies. Sodium methoxide was first prepared by dissolving NaOH in methanol and then mixed with preheated *Camelus dromedarius* oil in a three-neck flask. The reaction mixture was maintained at the desired temperature for the specified duration. A stirring speed of 350 rpm was employed to ensure efficient mixing between the immiscible oil and methanol phases, thereby enhancing mass transfer and promoting effective contact between the reactants and catalyst throughout the transesterification process (Nguyen et al, 2026). Upon completion, the mixture was transferred to a separation funnel, where it was separated into two distinct layers: biodiesel (upper) and glycerol (lower). The biodiesel layer was carefully separated and purified by repeated wet washing with 50 mL portions of warm distilled water (50–60 °C) at a 1:1 (v/v) biodiesel-to-water ratio until the wash water became clear to remove residual catalyst, glycerol, and excess methanol.^4,18,28^ The washing process was repeated until the wash water became clear (three washing cycles), indicating effective removal of impurities. After washing, the biodiesel was dried by heating at 105 °C for 3 h to remove residual moisture. Drying continued until no visible moisture remained or until a constant mass was achieved. Finally, the biodiesel yield was calculated using eq 2

2 $$\mathrm{Biodiesel yield}\left(\%\right)=\frac{W_{b}}{W_{0}}\times 100$$

where:

**Figure 1:**
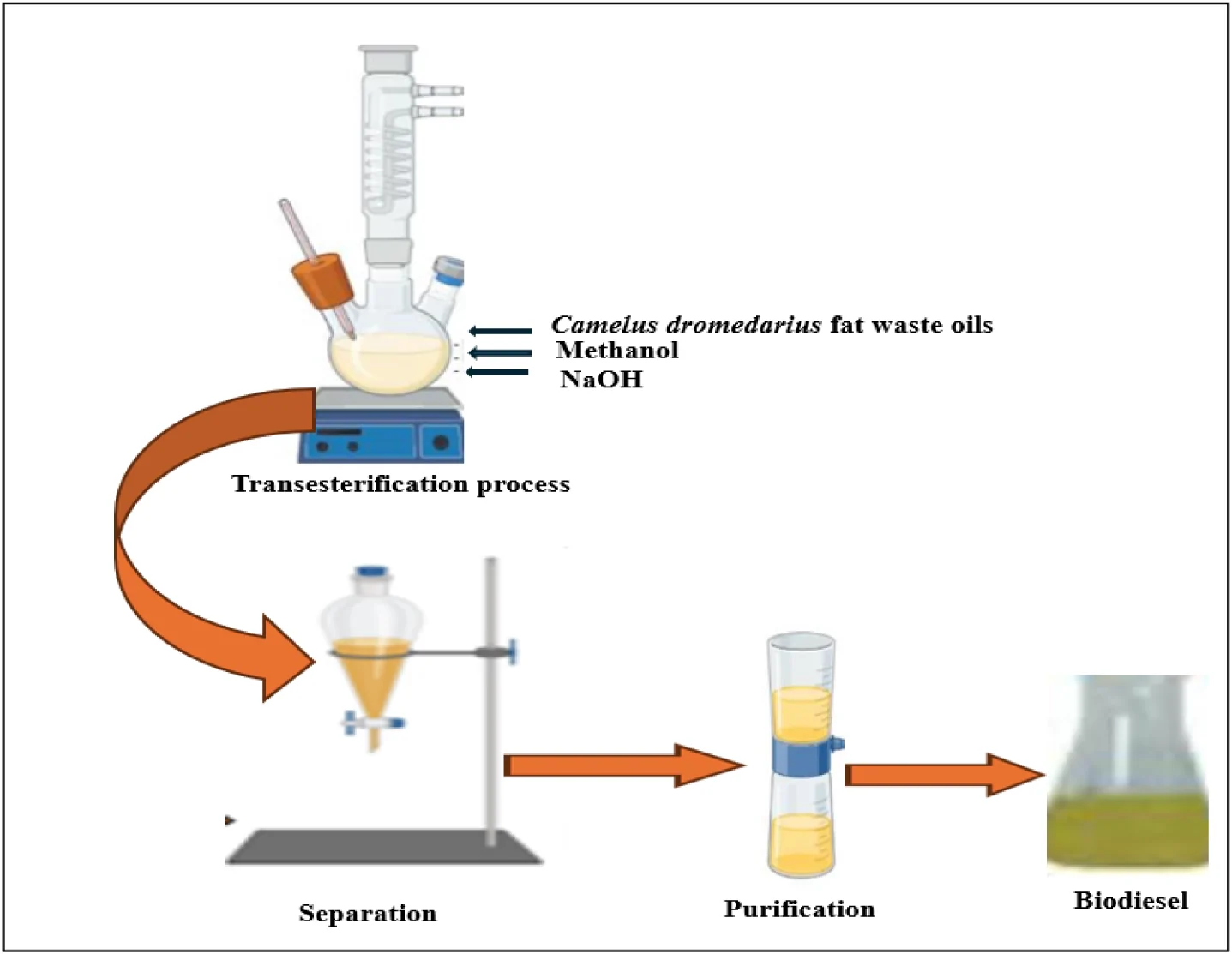
Schematic representation of the transesterification process for biodiesel production from camel fat waste.

**Table 1:** Ranges of Operational Parameters for Biodiesel Production

Transesterification parameters	Unit	Range
Oil-to-methanol molar ratio	(mol/mol)	1:3–1:12
Catalyst ratio	(wt %)	0.25–1
Reaction temperature	(°C)	55–70
Reaction time	(min)	15–60

• *W_b_* = weight of biodiesel produced (g)

• *W*_0_= weight of the extracted oil used for transesterification (g)

### 2.3 RSM Design of Transesterification Experiment

The transesterification process for biodiesel production from *Camelus dromedarius* fat waste was optimized using RSM based on a Central Composite Design (CCD). The experimental design considered four independent variables: oil-to-methanol molar ratio (A), catalyst concentration (B), reaction temperature (C), and reaction time (D), while biodiesel yield (%) was taken as the response variable (Y). The selection of these parameters was supported by previous studies that identified them as key factors influencing transesterification efficiency.^4,29^ The ranges of these variables were established from preliminary one-factor-at-a-time (OFAT) experiments. The ranges presented in Table 1 correspond to the preliminary OFAT screening experiments used to identify suitable operating regions for biodiesel production. For the subsequent CCD-RSM optimization, broader factor limits were selected (Table 2) to adequately evaluate quadratic effects and interactions among variables. Therefore, the ranges reported in Table 2 represent the actual coded levels employed in the optimization design. Each independent variable influencing *Camelus dromedarius* biodiesel production was examined at three coded levels: low (−1), medium (0), and high (+1). The CCD employed in this study consisted of 30 experimental runs, including 16 factorial points, 8 axial points, and 6 center-point replications. The axial distance (α) was set at 2.0 to ensure rotatability of the design. Experimental runs were randomized to minimize systematic bias and reduce the influence of uncontrolled variables. The coded levels shown in Table 2 correspond to the actual factor levels used in the experiments.

**Table 2:** Coded Levels of the Independent Variables for Biodiesel Production

			Levels		
No	Independent variables/unit	Symbol	–1	0	+1
**1**	Oil-to-methanol molar ratio (mol/mol)	A	1:3	1:9	1:15
**2**	Catalyst ratio (wt %)	B	0.25	0.75	1.25
**3**	reaction Temperature (°C)	C	45	65	85
**4**	Reaction Time (min)	D	15	35	55

A second-order polynomial model was employed, as it provides an accurate representation of curvature in the response and captures interaction effects between variables, which are significant in biodiesel synthesis. The transesterification process was expressed mathematically in eq 3.

3 $$Y=b_{0}+\overset{n}{\underset{i=1}{\sum }}b_{i}X_{i}+\overset{n}{\underset{i=1}{\sum }}b_{\mathrm{ii}}X_{i}^{2}+\overset{n}{\underset{i<j}{\sum }}b_{\mathrm{ij}}X_{i}X_{j}$$

where Y represents the predicted *Camelus dromedarius* biodiesel. The model coefficients from the regression model include B_i_, B_ii_, and B_ij_. They indicate the linear, quadratic, and interactions of the independent factors and their effect on result response. X_i_ and X_j_ represent the independent variables. Lastly, *n* stands for the coded variables in the polynomial equation.

The adequacy of the model fit was validated by examining the regression coefficients: the coefficient of determination (R^2^), adjusted R^2^, and predicted R^2^. The statistical significance of the overall model was assessed using Analysis of Variance (ANOVA), which included evaluating p-values and performing a lack-of-fit test to confirm the model’s robustness and predictive capability. Response surface plots were generated to visually interpret the interactions between variables and identify optimal conditions for maximum biodiesel yield. All experimental design, analysis, and plot generation were carried out using Design-Expert 11 software. Each experimental run in the design was conducted in triplicate under identical operating conditions, and the average biodiesel yield together with the corresponding standard deviation was calculated.^11,30,31^

### 2.4 Characterization of Extracted Oil and Biodiesel

The extracted *C. dromedarius* fat waste oil and the synthesized biodiesel were characterized based on their chemical composition and physicochemical properties to evaluate their quality and suitability for biodiesel production.

Gas chromatography with a flame ionization detector (GC-FID) to determine the fatty acid composition. 50 mg of the sample was dissolved in *n*-hexane and injected into the GC-fid system. The column used was a 30-m capillary column coated with a 0.25 μm film. The column temperature was initially set at 150 °C, with a ramp rate of 5 °C/min up to 195 °C. The injector and detector temperatures were set at 200 and 220 °C, respectively. The GC-FID system was calibrated using a Supelco 37 Component FAME Mix (Sigma-Aldrich), and individual fatty acid methyl esters were identified by comparing their retention times with those of the corresponding standard mixture. Fatty acid composition was determined from the chromatographic peak areas and expressed as the percentage of the total identified FAMEs.

Moreover, Fourier transform infrared (FTIR) spectroscopy was utilized to confirm the functional group of the extracted oil into esters. A PerkinElmer System 2000 FTIR was used to analyze the samples. This spectrometer features a detector with a spectral range from 4000 to 600 cm-1 and a resolution of 5 cm-1. The Nicolet OMNIC 5.01 software was used to process the IR spectra obtained from the spectrometer.^11,32^

Furthermore, physicochemical properties were assessed according to internationally accepted standards, as presented in Table 3. They were verified using established methods from recent studies to ensure precision and comparability. Density at 15 °C was determined using the hydrometer method in accordance with ASTM D1298,^33^ while kinematic viscosity was measured at 40 °C using a capillary viscometer following ASTM D446 ^34^ The cloud point and pour point were determined through the test jar method in compliance with ASTM D2500 ^35^ and ASTM D97 ^36^ respectively. The cetane number was experimentally determined using a calibrated Cooperative Fuel Research (CFR) engine in accordance with ASTM D613.^37^ The engine was operated under standardized conditions at 900 rpm, and the cetane number was determined by comparing the ignition delay of the test sample with reference fuels of known cetane numbers through interpolation. Thermal properties, including flash point and heating value, were assessed with a Pensky-Martens closed-cup apparatus and a bomb calorimeter, in accordance with ASTM D93 ^38^ and ASTM D240 ^39^ respectively. Additionally, titration analyses against KOH, HCl, and sodium thiosulfate were conducted to ascertain acid value, saponification value, and iodine number, following ISO 660:2009,^40^ AOAC 920.160,^41^ and AOAC 920.159,^42^ respectively. Finally, the moisture content of the extracted oil was determined in accordance with ASTM D6304 (2020) using the Coulometric Karl Fischer titration method. All experimental procedures were carried out in triplicate, and the results are presented as mean values with associated standard deviations (SD).^26,43–47^

**Table 3:** Standard Test Methods for Physicochemical Properties

Physicochemical properties	Standard method
Density at 15 °C	ASTM D1298
Kinematic viscosity at 40 °C	ASTM D446
Pour point	ASTM D97
Cloud point	ASTM D2500
Heating value	ASTM D240
Flash point	ASTM D93
Cetane number	ASTM D613
Acid value	IOS 660:2009
Saponification value	AOAC 920.160
Iodine value	AOAC 920.159
Moisture content	ASTM D6304 (2020)

## 3 Results and Discussion

### 3.1 Oil Extraction

The highest oil yield obtained from the *Camelus dromedarius* fat waste was 70.10%, achieved under temperature of 70 °C, a fat-to-solvent (F/S) ratio of 1:10 mol/mol, and a treatment duration of 5 h using the Soxhlet extraction method. For comparison, Mat Yasin et al.,^48^ employing the same Soxhlet approach, reported a lower lipid yield of 59.92% from fish fat, despite applying more severe conditions of 85 °C, 5 h, and a 1:2 F/S ratio. In a separate study, Aminullah et al.^49^ evaluated different solvent extraction techniques for beef fat waste, ranking the methods in terms of lipid extraction as Soxhlet (89.67%), Hara and Radin (84.40%), Folch (81.73%), and Bligh and Dyer (55.16%). Additionally, Jambulingam et al. reported a comparatively lower extraction yield of 48.35% from discarded beef waste using autoclave-assisted extraction at 120 °C and 2 bar.^50^

### 3.2 Assessment of Transesterification Factors

#### 3.2.1 Effect of Oil-to-Methanol Molar Ratio

The oil-to-methanol molar ratio is a critical parameter influencing the transesterification process for biodiesel production from *Camelus dromedarius* fat waste oil. In this study, the molar ratio was assessed from 1:3 to 1:12 mol/mol at 60 °C, 45 and 0.5 wt %, constant reaction temperature, reaction time, and catalyst ratio. This is shown in Figure 2a. The biodiesel yield increased with higher methanol ratios up to an optimal point. The yield was 64.24% at 1:3 mol/mol and rose to 81.12% at 1:6 mol/mol, reaching a maximum of 94.60% at 1:9 mol/mol. Therefore, an oil-to-methanol ratio of 1:9 mol/mol was identified as the most effective for achieving maximum biodiesel yield from *Camelus dromedarius* fat waste oil. Beyond this point, the yield gradually declined at 1:12 mol/mol. This decline at excessive methanol levels occurs because high alcohol concentrations dilute the fatty acids, such as oleic acid, and increase the solubility of glycerol in the ester phase. This promotes partial reversibility of the transesterification reaction and leads to the formation of monoglycerides and diglycerides, ultimately reducing the overall yield to fatty acid methyl esters.^51^ Similar findings have been reported in previous studies, where the biodiesel yield increased with the molar ratio up to an optimum of approximately 1:9, beyond which a decline in yield was.^18,52^

**Figure 2:**
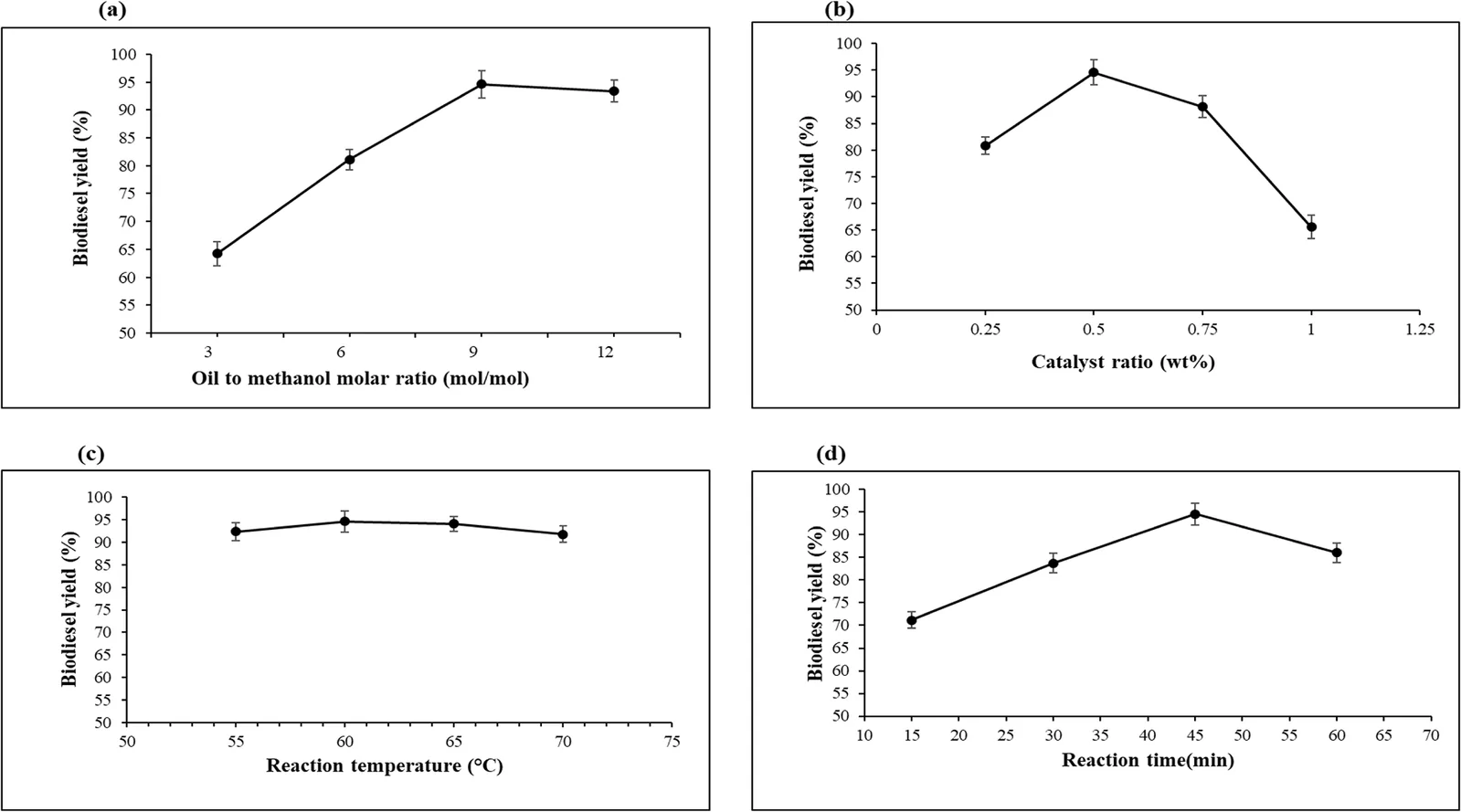
Assessing transesterification factors: (a) Effects of oil-to-methanol molar ratio on transesterification reaction; (b) effects of catalyst ratio on transesterification reaction; (c) effects of reaction temperature on transesterification reaction; (d) effects of reaction time on transesterification reaction.

#### 3.2.2 Effect of Catalyst Ratio

The transesterification of *Camelus dromedarius* fat waste oil into biodiesel was carried out using NaOH as a strong base catalyst. Base catalysts can accelerate the transesterification reaction up to 400 times faster than acid catalysts, making them highly effective for biodiesel production.^53^ In this study, the NaOH ratio significantly affected the transesterification process. The effect of catalyst ratio on biodiesel yield was investigated at constant conditions: an oil-to-methanol molar ratio of 1:9 mol/mol, a temperature of 60 °C, and a reaction time of 45 min as shown in Figure 2b. There is an increase in biodiesel yield with an increase in catalyst ratio. The highest yield of 94.60% was obtained at 0.50 wt %. Increasing the catalyst ratio above 0.5 reduced the yield. The decrease at higher catalyst loadings is attributed to soap formation (saponification), which hinders phase separation and lowers the production efficiency of *Camelus dromedarius* oil into biodiesel.^54^ Similar behavior has been reported in previous studies, where biodiesel yield improved with catalyst loading up to an optimum typically between 0.5 and 1.0 wt %.^4,55^

#### 3.2.3 Effect of Reaction Temperature

Temperature is a vital parameter influencing the transesterification process for converting *Camelus dromedarius* fat waste oil into biodiesel. In this study, the process was conducted at temperatures ranging from 55 to 70 °C. Other factors were kept constant: an oil-to-methanol molar ratio of 1:9 mol/mol, a catalyst ratio of 0.5 wt %, and a reaction time of 45 min. The effect of reaction temperature on the ester content is shown in Figure 2c. There was an increase in the yield of biodiesel, which increased from 55 to 60 °C. The highest yield of 94.60% was obtained at 60 °C. Beyond this temperature, the yield was declined to 94.10% at 65 °C and further to 91.80% at 70 °C. The initial increase in yield with rising temperature can be attributed to enhanced reaction kinetics, which accelerate the transesterification process. However, temperatures above 60 °C likely promote methanol evaporation, reducing the effective methanol concentration and shifting the reaction equilibrium unfavorably, thereby decreasing biodiesel yield. In addition, elevated temperatures may enhance saponification in the presence of NaOH leading to soap formation. This side reaction consumes part of the catalyst, hinders the separation of biodiesel and glycerol, and consequently reduces the overall biodiesel yield.^54^

#### 3.2.4 Effect of Reaction Time

Reaction time significantly influences biodiesel production from *Camelus dromedarius* fat waste oil. In this study, the effect of reaction time on biodiesel yield was evaluated at varying time from 15 to 60 min. The effect of reaction time on biodiesel yield was examined at 1:9, 0.5 wt %, and 60 °C, constant an oil-to-methanol molar ratio, a catalyst ratio, and temperature, as shown in Figure 2d. As time was increased, an increase in the yield was also observed; this rise in yield is because there was enough time for the reaction to occur. The maximum yield 94.60% was obtained at 45 min. Extending the reaction time to 60 min reduced the yield to 86.10%. The initial increase occurs as more triglycerides are converted into methyl esters with longer reaction durations. However, excessive reaction time promotes reverse reactions and methanol loss, decreasing production efficiency. This indicates that beyond an optimal point, prolonged reaction does not further enhance biodiesel yield^54^

Overall, the observed response surfaces indicate that biodiesel production from *C. dromedarius* fat waste is governed by a combination of chemical reaction kinetics and mass transfer phenomena. Under lower operating conditions, reaction rates are limited by insufficient methanol availability, catalyst activity, and interfacial mass transfer between the immiscible reactants. As these parameters approach their optimum values, reaction kinetics become increasingly favorable, leading to enhanced triglyceride conversion to methyl esters. Beyond the optimum region, however, competing phenomena, such as methanol dilution effects, saponification, glycerol solubility, and equilibrium limitations, reduce the overall biodiesel yield. These findings demonstrate that achieving high biodiesel conversion requires balancing reaction kinetics with process conditions that minimize undesirable side reactions.^56^

### 3.3 Optimization of Transesterification Process

#### 3.3.1 Analysis of RSM Model

RSM was applied to evaluate the relation between the response *C. dromedarius* biodiesel and reaction variables, namely, oil-to-methanol molar ratio, catalyst ratio, reaction time, and reaction temperature. This approach enables efficient modeling and analysis of multiple influencing variables while reducing the number of experimental trials required. By optimizing experimental conditions, RSM helps save time and resources and enhances the accuracy of predictions, making it a crucial step in improving biodiesel production.^57^ In this study, a central composite (CCD) within the RSM model was employed to develop a second-order polynomial regression model for the transesterification process. This model enabled the assessment of both individual and interactive effects of the process variables on the biodiesel yield.

The adequacy and reliability of the developed model were assessed using analysis of variance (ANOVA). This statistical approach determines whether the model and its terms significantly influence the response variable by analyzing the p-values. For a model to be considered statistically significant, the p-value should be less than 0.05, whereas a p-value greater than 0.05 for the lack of fit indicates that the model fits the data reasonably well within experimental error. However, it should be noted that a borderline lack-of-fit value requires a cautious interpretation of the model performance. Additionally, the model performance metrics, including R^2^ (0.9915), adjusted R^2^ (0.9836), and predicted R^2^ (0.9548), presented in Table 4, demonstrate strong statistical performance with good predictive consistency within the studied range.

**Table 4:** Statistical Indicators Derived from RSM for the Transesterification of *Camelus dromedarius* Fat Waste to Biodiesel

Variables	Value
R^2^	0.9915
Adjusted R^2^	0.9836
Adequate Precision	40.7666
SD	0.3645
Mean	90.30
Coefficient of variations %	0.4037
Predicted R^2^	0.9548

The analysis of variance (ANOVA) in Table 5 confirms that the model is significant, evidenced by a p-value <0.0001. However, the lack-of-fit test was found to be borderline (p = 0.0540), indicating that although the model is statistically adequate, minor unexplained variations may exist, and the results should be interpreted with some caution. In addition, the interaction term CD was statistically insignificant (*p* > 0.05), suggesting that the combined effect of catalyst ratio and reaction time has a negligible influence on biodiesel yield. Therefore, based on the regression analysis, a second-order polynomial equation as shown in eq 4 was established to describe the relationship between biodiesel yield and the coded values of the independent variables.

**Table 5:** Analysis of Variances (ANOVA) of the Fitted Model for the Transesterification to synthesize*Camelus dromedarius* Biodiesel

Sources	Sum of square	Degree of freedom	Mean square	F-value	p-Value	Significance
Model	233.45	14	16.67	125.48	<0.0001	significant
A	17.17	1	17.17	129.21	<0.0001	
B	7.82	1	7.82	58.85	<0.0001	
C	9.13	1	9.13	68.68	<0.0001	
D	0.9600	1	0.9600	7.22	0.0169	
AB	1.00	1	1.00	7.53	0.0151	
AC	3.24	1	3.24	24.38	0.0002	
AD	6.00	1	6.00	45.17	<0.0001	
BC	7.43	1	7.43	55.88	<0.0001	
BD	1.76	1	1.76	13.21	0.0024	
CD	0.2256	1	0.2256	1.70	0.2122	
A^2^	133.26	1	133.26	1002.77	<0.0001	
B^2^	31.57	1	31.57	237.60	<0.0001	
C^2^	24.32	1	24.32	183.02	<0.0001	
D^2^	48.46	1	48.46	364.65	<0.0001	
Residual	1.99	15	0.1329			
Lack of Fit	1.80	10	0.1796	4.56	0.0540	not significant
Pure Error	0.1971	5	0.0394			
Cor Total	235.44	29				

The lack-of-fit test was not statistically significant (*p* = 0.0540), indicating that the developed quadratic model adequately represents the experimental data within the investigated design space. However, since the lack-of-fit *p*-value is only marginally higher than the significance level of 0.05, the model should be regarded as statistically acceptable rather than a perfect representation of the transesterification process. This borderline lack of fit suggests that a small amount of unexplained variability may still exist, which may be attributed to inherent experimental variability or other minor factors not included in the current model. Nevertheless, the high coefficient of determination (R^2^ = 0.99), together with the good agreement between the predicted and experimental biodiesel yields, demonstrates that the model possesses a satisfactory predictive capability for process optimization.

The residual mean square value was found to be 0.19%, indicating acceptable agreement between experimental and predicted values within experimental error, as detailed in Table 6. Additionally, Figure 3 illustrates the agreement between the actual and predicted responses alongside the normal probability plot of residuals. The close alignment of data points along the ideal line indicates reasonable model accuracy and predictive capability rather than perfect agreement. Supported by the data presented in Table 7, the analysis results suggest that the model adequately represents the observed experimental data within the investigated design space for the transesterification of *Camelus dromedarius* fat waste into biodiesel.

4 $$Y=94.74+0.8458A+0.5708B+0.6167C+0.20D+0.\mathrm{25AB - 0.45AC}+0.\mathrm{6125AD}-0.\mathrm{6812BC}+0.\mathrm{3313BD}-2{.2A}^{2}\mathrm{- 1}{.07B}^{2}-0{.9417C}^{2}-1{.33D}^{2(1)}$$

where Y represents the *Camelus dromedarius* biodiesel yield (%), and A, B, C, and D, are the coded values of oil-to-methanol molar ratio, catalyst ratio, temperature, and time, respectively.

**Table 6:** Regression Coefficients and the Statistical Significance of the Quadratic Model for the Transesterification to Synthesize *Camelus dromedarius* Biodiesel

				Confidence level	
Factor	Coefficient estimate	Degree of freedom	Standard error	Low	High
Intercept	94.74	1	0.1488	94.42	95.06
A	0.8458	1	0.0744	0.6872	1.00
B	0.5708	1	0.0744	0.4122	0.7294
C	0.6167	1	0.0744	0.4581	0.7753
D	0.2000	1	0.0744	0.0414	0.3586
AB	0.2500	1	0.0911	0.0558	0.4442
AC	–0.4500	1	0.0911	–0.6442	–0.2558
AD	0.6125	1	0.0911	0.4183	0.8067
BC	–0.6812	1	0.0911	–0.8755	–0.4870
BD	0.3313	1	0.0911	0.1370	0.5255
CD	0.1188	1	0.0911	–0.0755	0.3130
A^2^	–2.20	1	0.0696	–2.35	–2.06
B^2^	–1.07	1	0.0696	–1.22	–0.9246
C^2^	–0.9417	1	0.0696	–1.09	–0.7933
D^2^	–1.33	1	0.0696	–1.48	–1.18

**Table 7:** Comparison of Actual and Predicted Responses of the Transesterification Reaction to Produce *Camelus dromedarius* Biodiesel

Run	Variables				Responses	
	A	B	C	D	Actual	Predicted
1	0	0	0	0	94.6	94.74
2	1	1	1	–1	89.25	89.08
3	1	1	1	1	91.25	91.61
4	1	–1	–1	–1	88.3	88.01
5	0	0	0	0	94.6	94.74
6	0	–2	0	0	89.25	89.31
7	1	1	–1	–1	90.1	90.35
8	0	0	0	0	94.85	94.74
9	–2	0	0	0	84	84.23
10	0	0	0	0	95.1	94.74
11	0	0	2	0	92.55	92.21
12	–1	–1	1	1	89.5	89.15
13	0	0	–2	0	89.45	89.74
14	0	0	0	–2	88.7	89.03
15	–1	1	1	1	88.65	89.09
16	2	0	0	0	87.9	87.62
17	0	0	0	2	90.2	89.83
18	–1	–1	–1	1	85.1	85.42
19	1	1	–1	1	92.45	92.40
20	–1	–1	–1	–1	87.6	87.14
21	0	0	0	0	94.65	94.74
22	–1	1	–1	1	88.35	88.08
23	–1	1	1	–1	89.2	89.02
24	0	0	0	0	94.65	94.74
25	–1	1	–1	–1	88.6	88.48
26	0	2	0	0	91.7	91.59
27	1	–1	–1	1	88.65	88.73
28	–1	–1	1	–1	90.2	90.40
29	1	–1	1	–1	89.3	89.47
30	1	–1	1	1	90.4	90.67

**Figure 3:**
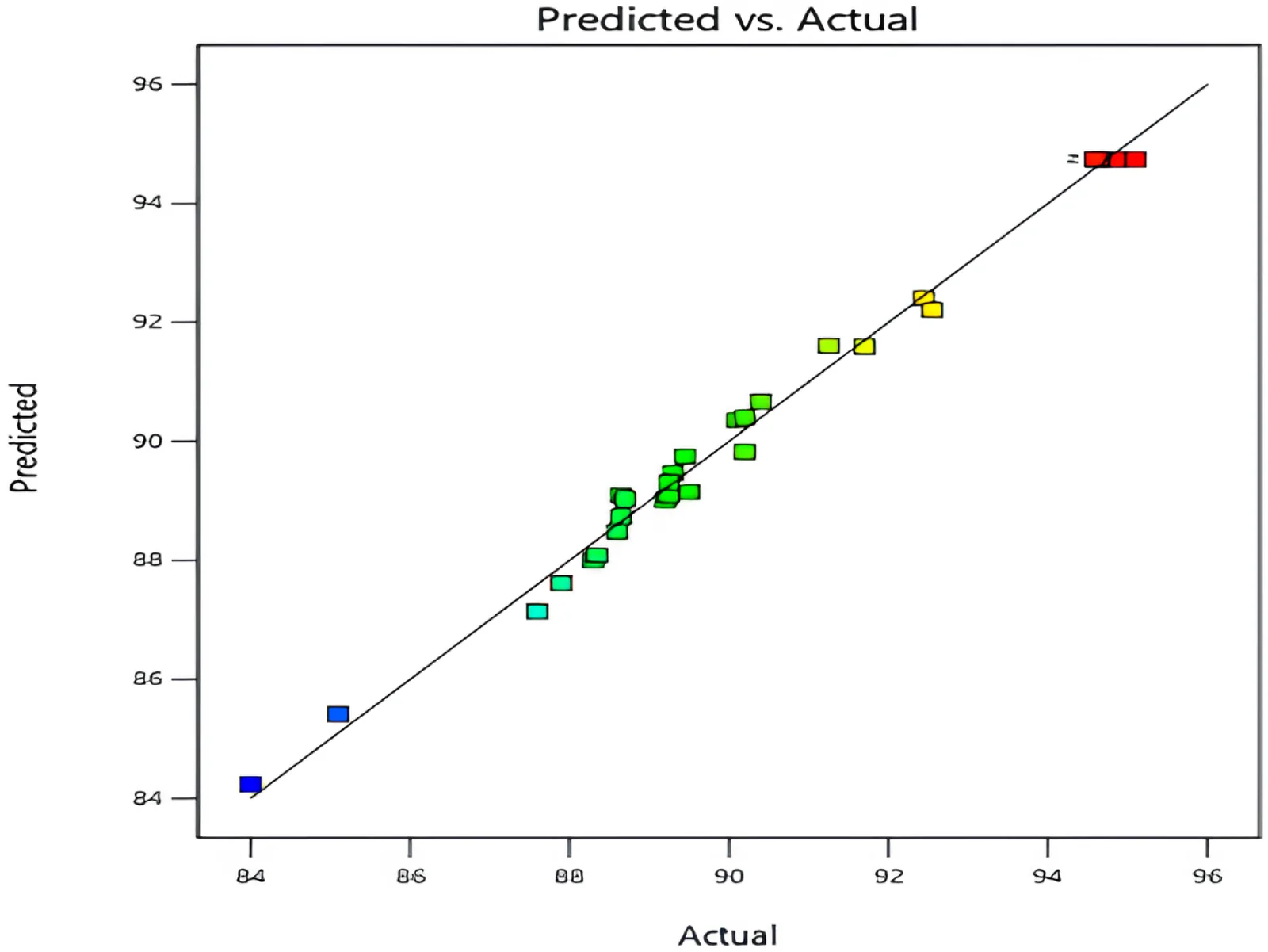
Spread of actual outcomes and predicted values from the transesterification reaction.

The transesterification process for converting *Camelus dromedarius* fat waste into biodiesel was optimized to maximize yield. The optimized biodiesel yield with its standard deviation based on 3 replicates was obtained from operational parameters of transesterification of 95 ± 1.50%. This peak yield corresponded to the following reaction conditions: oil-to-methanol molar ratio of 9.6:1 mol/mol, a NaOH catalyst loading of 0.8 wt %, a reaction temperature of 68 °C, and a reaction time of 36 min as presented in Table 7. Since the optimum reaction temperature 68 °C is close to the boiling point of methanol, the transesterification reaction was performed in a sealed reactor to prevent methanol volatilization at the reaction temperature. The reactor was fitted with a condenser to allow the reflux of evaporated methanol back into the reaction mixture, thereby maintaining the pressure and concentration throughout the reaction.

To verify the model’s predictive accuracy, three independent experiments were conducted using these optimal settings. The average experimental yield was 94.82% with the smallest relative error recorded at 0.19%. These findings confirm the strong predictive capability of the RSM employed in modeling in the transesterification reaction for producing biodiesel from *Camelus dromedarius* fat waste. The model was validated experimentally under the optimum operating conditions with a relative error of only 0.19%. To further assess the adequacy and robustness of the developed quadratic model, diagnostic plots, including residuals versus predicted values, leverage analysis, and influence diagnostics, were examined. The residuals were randomly scattered around zero without any discernible pattern, indicating constant variance and the absence of a systematic model bias. The leverage and influence analyses showed that no experimental run exerted an undue influence on the regression model, confirming that the developed model was statistically robust and suitable for predicting the biodiesel yield within the investigated experimental domain. A comparative summary of the present study optimized parameters alongside previous research utilizing various animal fat feedstocks is presented in Table 8.

**Table 8:** Optimum Values of Transesterification Reactions to Synthesize Biodiesel from *Camelus dromedarius* Fat Waste

Transesterification parameters	Unit	Optimum values
Oil-to-methanol molar ratio	mol/mol	1:9.6
Catalyst ratio	Wt%	0.8
Reaction time	Min	36
Reaction temperature	°C	68
Biodiesel yield	%	95

The optimum conditions for the transesterification of synthesized biodiesel fuel from *Camelus dromedarius* waste fat, as obtained in the present study, were compared with those reported in previous studies, as presented in Table 9. The biodiesel yield of 95% achieved in this study was higher than the 89% reported by Mohammed et al.,^23^ who utilized fish oil under the conditions of a 4.75:1 mol/mol oil-to-methanol molar ratio, 0.87 wt % KOH, 60 min of reaction time, and a reaction temperature of 50 °C. Similarly, Shalfoh et al.,^29^ reported a 92% biodiesel yield from fish waste oils using a 6:1 mol/mol oil-to-methanol molar ratio, 2 wt % NaOH catalyst, a reaction temperature of 60 °C, and a reaction time of 90 min. In addition, the findings of Binhweel et al.^4^ achieved a comparable biodiesel yield of approximately 95.15% from discarded beef tallow, although this required a longer reaction time of 72 min and a higher catalyst loading of 1.16 wt % KOH. Furthermore, Suwannapa and Tippayawong^58^ reported a 99% yield from waste beef tallow using microwave-assisted transesterification, which significantly differs in method and energy input, although it employed a similar temperature of 60 °C and a lower catalyst concentration of 0.62 wt %. Notably, the use of mixed lamb fat and beef tallow with a blast furnace geopolymer catalyst by Mwenge et al.^59^ achieved a 95% yield using a mixture of lamb fat and beef tallow with a blast furnace slag-based geopolymer catalyst, but under a higher catalyst loading of 1.5 wt % and a longer reaction time of 60 min.

**Table 9:** Comparison of the Optimum Values of the Present Study with Previous Studies Using Different Animal Fats as Feedstocks for Biodiesel

	Alcohol		Catalyst									
Feedstock	Alcohol type	Molar ratio(mol/mol)	Catalyst type	Catalyst ratio (wt %)	Reaction time (min)	Reaction temperature (°C)	Biodiesel yield (%)	Reaction mechanism	Statistical method	Energy demand	R^2^	Reference
*Camelus dromedarius* waste fat	Methanol	1:9.6	NaOH	0.8	36	68	95	Homogeneous base-catalyzed transesterification	RSM-CCD	Conventional heating	0.9915	Current study
Discarded Beef tallow	Methanol	1:7.5	KOH	1.16	72	58	95.15	Homogeneous base-catalyzed transesterification	RSM-CCD	Conventional heating	0.9990	^4^
Waste beef tallow	Methanol	1:9	NaOH	0.62	25	60 (microwave)	99	Homogeneous base-catalyzed transesterification	RSM (FFD + CCD)	Microwave heating	0.804	^58^
Fish waste oil	Methanol	1:6	NaOH	2	90	60	92.1	Homogeneous base-catalyzed transesterification	RSM-CCD	Conventional heating	0.9981	^29^
Fish waste oil	Methanol	4.75:1	KOH	0.87	60	50	89.0	Homogeneous base-catalyzed transesterification	RSM (CCD)	Conventional heating	-	^23^
Mixed lamb fat and beef tallow	Methanol	1:9	Blast furnace	1.5	60	65	95.0	Heterogeneous base-catalyzed transesterification	RSM-CCD	Conventional heating	0.9465	^59^
Fish waste oil	Methanol	4.7:1	ZnO	13.2	-	33	91	Heterogeneous base-catalyzed transesterification	RSM-CCD	Conventional heating	0.9754	^60^

#### 3.3.2 Effects of Independent Variables

The influence of transesterification process parameters, both as individual factors and through their interactive effects, on the biodiesel yield derived from *Camelus dromedarius* fat waste was investigated using the RSM approach. The selected process variables included the oil-to-methanol molar ratio, catalyst concentration, reaction time, and reaction temperature. These were compared in Figure 4. based on their effects on the percentage of biodiesel yield. To visualize the relationships and interactions among these independent variables and their influence on the biodiesel yield, three-dimensional (3D) response surface plots were generated and analyzed. The six pairwise interactions among the transesterification parameters exhibited a statistically significant influence on the biodiesel yield, highlighting the complex interdependence of process conditions in optimizing biodiesel production from *Camelus dromedarius* fat waste.

**Figure 4:**
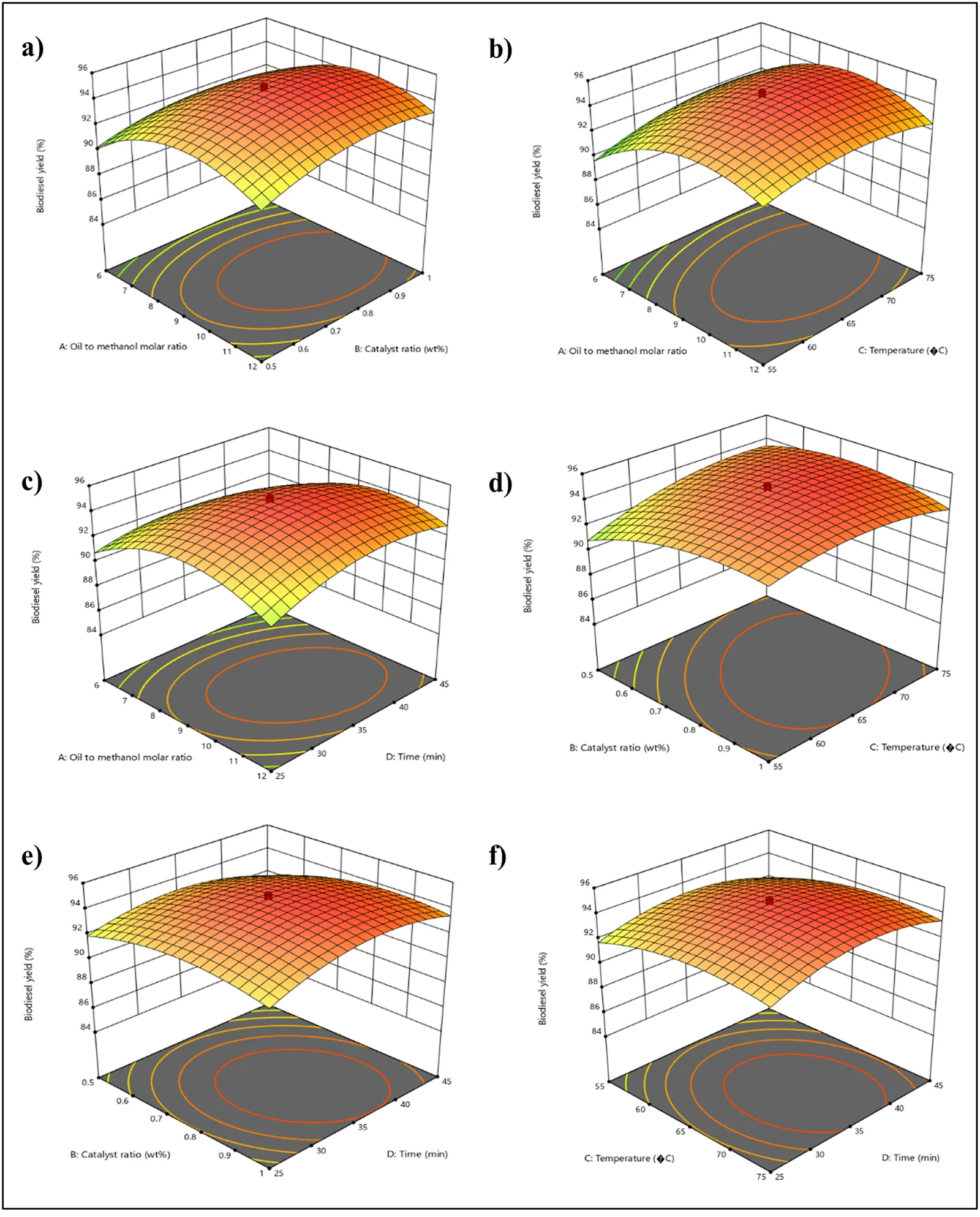
3D surface plots describe the interaction impacts of the independent variables on biodiesel yield response. (a) oil- to- methanol molar ratio and catalyst amount (b) oil- to- methanol molar ratio and temperature (c) oil- to- methanol molar ratio and time (d) catalyst amount and temperature (e) catalyst amount and time (f) temperature and time.

The response surface plots presented in Figure 4 complement the ANOVA results by illustrating the combined influence of the process variables on biodiesel yield. The significant interaction terms (AB, AC, AD, BC, BD, and CD) indicate that the effect of one process variable depends on the level of another rather than acting independently. The curved response surfaces and elliptical contour plots confirm the presence of these interactions and demonstrate that simultaneous optimization of the transesterification parameters is necessary to achieve a maximum biodiesel yield. Among the investigated variables, the oil-to-methanol molar ratio exhibited the strongest interaction with the other process variables, whereas the reaction time showed comparatively weaker interaction effects, consistent with the statistical analysis presented in the regression model.

Figure 4a illustrates the interaction between oil-to-methanol molar ratio (A) and catalyst concentration (B) on biodiesel yield, under constant conditions of 65 °C reaction temperature and 35 min reaction time. The plot shows that biodiesel yield increased with rising levels of both variables up to an optimum point, where the highest yield of approximately 94.8% was achieved at an oil-to-methanol ratio of 1:10 and a catalyst concentration near 0.8 wt %. This improvement occurred because a higher methanol ratio beyond the stoichiometric requirement drives the transesterification reaction toward ester formation, while increased catalyst concentration provides more active sites for the transesterification reaction, enhancing biodiesel (FAME) formation. However, excessive methanol can cause dilution effects and separation issues, whereas too much catalyst promotes soap formation through saponification, both of which slightly reduce yield beyond the optimal range.^61,62^

For the interaction between oil-to-methanol molar ratio and reaction temperature, Figure 4b shows that biodiesel yield increased with rising methanol ratio and temperature, reaching an optimum yield of about 94.8% at an oil-to-methanol ratio of approximately 1:10 and a temperature near 68 °C, while catalyst concentration and reaction time were kept constant at 0.75 wt % and 35 min, respectively. Beyond these levels, a slight decline in yield was observed. This trend can be attributed to the fact that higher methanol ratios initially shift the equilibrium toward methyl ester formation, while increased temperature accelerates the reaction rate and reduces viscosity, enhancing mass transfer. However, excessive methanol can cause separation issues, and temperatures above the optimal range may lead to methanol evaporation and possible side reactions, both of which can lead to reduced yields.^19,63^ Figure 4c illustrates the combined influence of the oil-to-methanol molar ratio and reaction time on biodiesel yield. An initial increase in both variables significantly enhanced biodiesel production, reaching an optimum yield of approximately 94–95% at around a 9:1 mol/mol molar ratio and 35 min reaction time. This improvement is due to greater methanol availability promoting the forward transesterification reaction and extended time allowing more complete biodiesel formation. However, beyond this optimum, further increases in either factor reduced yield. Excessive methanol can hinder phase separation and complicate glycerol removal, while prolonged reaction time can increase the likelihood of reverse reactions and soap formation, especially in the presence of residual FFAs. These trends were observed under constant conditions of 0.75 wt % catalyst concentration and 65 °C reaction temperature.^64,65^

Figure 4d illustrates the combined effect of catalyst ratio and reaction temperature on biodiesel yield at a fixed oil-to-methanol molar ratio of 9:1 and a reaction time of 35 min. The response surface shows that increasing the catalyst ratio from 0.5 to about 0.8 wt % and raising the temperature from 55 °C to around 70 °C significantly improved biodiesel yield, reaching an optimum of approximately 95%. Beyond these levels, a slight decline in yield was observed, likely due to soap formation at higher catalyst concentrations and methanol evaporation at elevated temperatures.^61^ Figure 4e shows the interaction between catalyst ratio and reaction time on biodiesel yield at a fixed oil-to-methanol molar ratio of 9:1 and a reaction temperature of 65 °C. The response surface reveals that increasing the catalyst ratio from 0.5 wt % to around 0.8 wt % and extending the reaction time from 25 to approximately 35 min significantly enhanced biodiesel yield, reaching an optimum near 95%. However, further increases in either parameter resulted in a slight decline in yield, which could be attributed to soap formation at excessive catalyst levels and possible reverse reactions or methanol loss with prolonged reaction time.^63^

Finally, Figure 4f displays the interaction between reaction temperature and reaction time on biodiesel yield under constant conditions of a 9:1 oil-to-methanol molar ratio and 0.75 wt % catalyst. The surface plot shows that increasing the temperature from 55 °C to about 70 °C and extending the reaction time from 25 to 35 min led to a progressive increase in biodiesel yield, reaching a peak near 95%. Beyond these optimal levels, further increases in either temperature or time caused a slight decline in yield, likely due to methanol evaporation at elevated temperatures and possible reverse reactions during prolonged reaction times.^66^ Collectively, the 3D surface plots in Figure 4a–f demonstrate that biodiesel yield from *Camelus dromedarius* fat waste is significantly influenced by the interaction of transesterification parameters. Among all variables, the methanol-to-oil molar ratio exhibited the most pronounced effect, while reaction time was the least influential factor.

### 3.4 Characterization

#### 3.4.1 FTIR Spectral Analyses

The IR spectra of *Camelus dromedarius* fat waste oil and the corresponding biodiesel are displayed in Figure 5. The characteristic functional groups of the extracted oil and resulting biodiesel were verified by the stretching and bending vibrations of C, H, and O bonds observed at various wavenumbers. The peak detected at 1741 cm^–1^ confirms the production of fatty acids into methyl esters, as it indicates the presence of the ester carbonyl group (C=O stretching). Additionally, strong peaks at 1172 cm^–1^ and 1197 cm^–1^ correspond to C–O stretching, further confirming the presence of ester groups. The absorption peaks at 2852 cm^–1^ and 2920 cm^–1^ are attributed to C–H stretching vibrations and O–H stretching vibrations, respectively. These peak positions closely agree with those reported for biodiesel produced from beef tallow, fish oil, and other waste animal fats, confirming the successful synthesis of biodiesel from *Camelus dromedarius* fat waste.^11,67^

**Figure 5:**
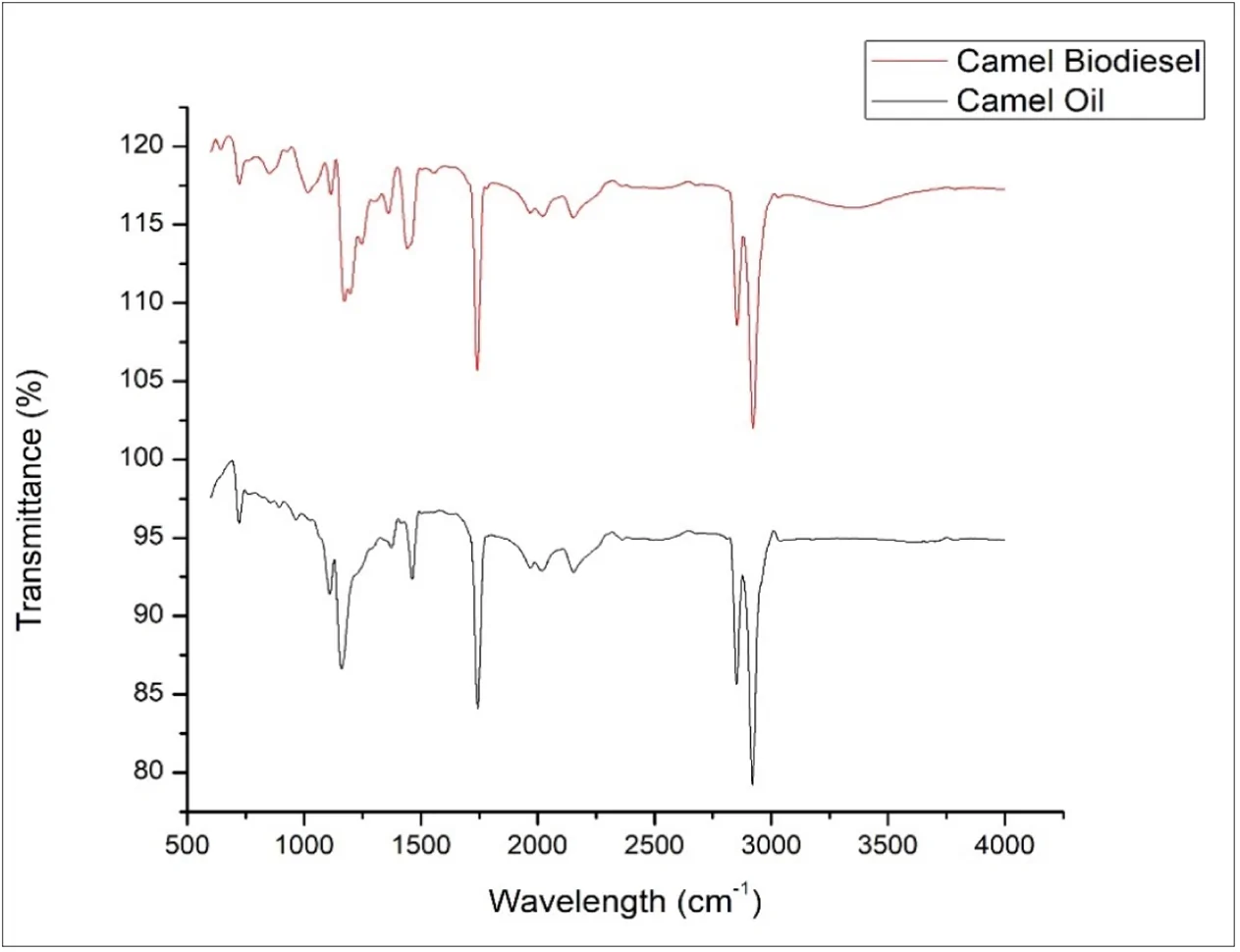
FTIR spectra of *Camelus dromedarius* fat waste oil and synthesized biodiesel.

#### 3.4.2 Fatty Acid Composition Analyses

Gas chromatography equipped with a flame ionization detector (GC-FID) was employed to comprehensively characterize the fatty acid profiles of camel dromedarius fat waste oil and the synthesized biodiesel. The detailed results are provided in Table 10. The extracted oil comprised eight long-chain fatty acids, C12–C20. Saturated fatty acids (SFAs) constituted nearly half of the total content (49.6%), with palmitic acid (C16:0) representing the largest fraction at 26.0%, followed by stearic acid (C18:0) at 11.2%. Unsaturated fatty acids (UFAs) accounted for 40.5%, predominantly oleic acid (C18:1), which contributed 31.3% to the total composition. These proportions are comparable to values reported for the same species in previous studies.^17^ The synthesized biodiesel contained a higher proportion of saturated fatty acids (SFAs) than unsaturated fatty acids (UFAs), as shown in Table 10. SFAs accounted for 53.7%, whereas UFAs represented 33.8%. The major sources of SFAs are palmitic acid (26.30%) and stearic acid (14.20%), which generally increase biodiesel cetane number, enhancing combustion efficiency and reducing emissions. However, their higher melting points can compromise cold-flow properties, potentially leading to fuel solidification and clogging.^10,68^ In contrast, USFAs such as oleic acid accounted for 25.20%. It improves cold-flow behavior, maintains low viscosity, and ensures reliable fuel flow under cold conditions, supporting efficient engine operability, preventing gelling or clogging, and improving low-temperature performance.^69,70^ The detailed fatty acid profile is critical for evaluating the biodiesel quality, performance, and oxidative stability, thus confirming its suitability as a sustainable alternative fuel.

**Table 10:** Fatty Acid Composition of *Camelus dromedarius* Oil and Synthesized Biodiesel

Fatty acid	C:D	Extracted oil (%)	Biodiesel (%)
Lauric	C 12:0	0.70	0.50
Myristic	C14:0	10.50	9.20
Myristoleic	C14:1	0.60	0.60
Pentadecanoic	C15:0	0.30	1.40
Palmitic	C16:0	26.0	26.30
Palmitoleic	C16:1	5.60	4.80
Margaric	C17:0	0.70	1.90
Heptadecenoic Acid	C17:1	0.80	2.00
Stearic	C18:0	11.20	14.20
Oleic	C18:1	31.30	25.20
Linoleic	C18:2	2.0	0.40
Linolenic	C18:3	0.20	0.40
Arachidic	C20:0	0.20	0.20
**Saturated fatty acids (SFAs)**		49.6	53.7
**Unsaturated fatty acids (UFAs)**		40.5	33.8

#### 3.4.3 Physicochemical Properties

The physicochemical properties of *C. dromedarius* fat waste oil and its biodiesel were determined based on density, kinematic viscosity, cloud point, pour point, flash point, heating value, acid value, saponification value, iodine value, and cetane number. The outcomes were compared with ASTM D6751 and EN 14214 standards. Table 11 presents the comparison of *Camelus dromedarius* oil and synthesized biodiesel characteristics with the biodiesel standards. In addition, multiple biodiesels derived from animal fat waste oil sources were compared with the results obtained in the current investigation in Table 12.

**Table 11:** Comparing the Physicochemical Properties of the *Camelus dromedarius* Fat Waste Oils and Synthesized Biodiesel with Biodiesel Standards

Physicochemical properties	Units	ASTM D6751	EN 14214	Diesel (ASTM D975)	Extracted oil	Synthesized biodiesel
Kinematic viscosity at 40 °C	mm^2^/s	1.9–6.0	3.5–5.0	1.9–4.1	16.31 ± 0.9	5.4 ± 0.2
Density at 15 °C	kg/m^3^	880	860–900	-	886 ± 12	877 ± 3
Heating value	MJ/kg	-	-	42–46	39.34±2.6	39.12 ± 2.5
Flash point °C	°C	Minimum 130	120 min	Minimum 52	200 ± 4.00	154 ± 2.00
Pour point	°C	–15 to −16	-	-	12 ± 1.20	9 ± 0.60
Cloud point	°C	–3 to −12	-	-	20 ± 1.50	18 ± 0.40
Acid value	Mg KOH/g %	Maximum 0.5	0.5 max	-	0.44 ± 0.05	0.11 ± 0.05
FFA	%	-	-	-	0.22 ± 0.05	0.056 ± 0.02
Iodine number	gmI^2^/100g	-	120 min	-	39.34 ± 2.5	31.84 ± 2.4
Saponification value	Mg KOH/g	Maximum 370	-	-	190.36 ± 4.2	184.66 ± 3.5
Moisture content	mg/kg	-	≤500	-	250 ± 8	430 ± 10
Cetane number	-	Minimum 47	51 min	Minimum 40	66.03 ± 0.19	68.69±0.57

**Table 12:** Comparison of the Biodiesel Properties Synthesized from Animal-Based Oils Feedstocks (Fish Waste, Chicken by-Products and Beef Tallow, and Poultry).^78,84^

				Biodiesel				
Physicochemical properties	Units	ASTM D6751	EN 14214	*Camelus dromedarius*	Poultry	Beef	Chicken fat	Fish fat
Kinematic viscosity at 40 °C	mm^2^/s	1.9–6.0	3.5–5.0	5.4 ± 0.2	6.86	5.35	4.9 ± 0.5	4.15 ± 0.1
Density at 15 °C	kg/m^3^	880	860–900	877± 3.5	877	870	869 ± 5	872.± 20
Flash point	°C	130 min	120 min	152± 2	172	171	171 ± 2.5	141 ± 20
Pour point	°C	–15 to −16	-	9±1.5	3	10	-2.08 ± 0.12	-7 ± 0.80
Cloud point	°C	–3 to −12	-	18±1	-	11±1.00	7 ± 10	3 ± 0.30
Acid value	Mg KOH/g %	0.5 max	0.5 max	0.11	0.55	0.20	0.28 ± 0.05	0.4 ± 0.10
Cetane number	-	47 min	51 min	68,69±1.2	-	62±0.48	-	55.16 ± 2.03
Heating value	MJ/kg	-	-	39.12±2.5	39.58	40.23	39.63 ± 1.21	39.2 ± 0.80
Iodine number	gmI^2^/100g	-	120 min	31.84±2.4	78.8	44.4	85.49 ± 1.84	91.6 ± 1.4
FFA	-	-	-	0.056	-	0.21±0.03	0.14 ± 0.01	0.20
Saponification value	Mg KOH/g	370 max	-	184.66±3.5	-	211±6.08	144 ± 1.5	158.54 ± 2.10
References				Current study	^11,85^		^26,86^	^29,87–89^

Density is a crucial property of biodiesel that influences engine performance and combustion behavior. It determines the delay between fuel injection and ignition in diesel engines and affects fuel delivery in volume-based injection.^71,72^ The synthesized biodiesel showed a density of 877 ± 3.5 kg/m^3^, which falls within the ASTM D6751 and EN 14214 standards. The density of biodiesel is strongly influenced by the composition of fatty acids, their molecular weights, and the presence of double bonds.^73^

Kinematic viscosity represents the resistance of a liquid to flow.^74^ Low viscosity facilitates fuel movement and improves atomization in diesel engines, reducing ignition delay.^75^ In contrast, excessively high viscosity can hinder fuel atomization, cause clogging in filters and pipes, reduce combustion efficiency, delay ignition, and increase harmful emissions.^45^ The kinematic viscosity of the *Camelus dromedarius* waste biodiesel was measured at 5.4 ± 0.2 mm^2^/s. The relatively high viscosity is primarily attributed to the elevated content of saturated fatty acids, particularly palmitic and stearic acids. Although this value satisfies the ASTM D6751 specification, it is slightly above the EN 14214 limit, which may adversely affect fuel atomization and produce larger fuel droplets during injection, resulting in less efficient combustion and increased injector deposits. Higher viscosity may also reduce cold-start performance under low-temperature conditions due to decreased fuel flow. However, the higher saturation level can contribute to improved oxidative storage stability. To improve compliance with EN 14214 and enhance fuel properties, blending camel fat biodiesel with lower-viscosity biodiesel or petroleum diesel is recommended.^55^

The cold-flow behavior of *Camelus dromedarius* oil and its biodiesel is largely governed by the fatty acid profile, including saturation level, chain length, mono- and polyunsaturated proportions, and the long-chain saturation factor. The synthesized biodiesel measured cloud and pour points of 18 ± 1 °C and 9 ± 1.5 °C, exceeding recommended biodiesel standards and indicating reduced operability at low temperatures. These higher values reflect the significant saturated fatty acid (SFA) content in the feedstock. Such poor cold-flow properties can cause gum formation and crystal deposition, potentially obstructing fuel lines, blocking pumps or injectors, and delaying engine start-up in colder conditions.^76^ To improve low-temperature performance, several strategies may be employed. Winterization can remove high-melting saturated components from the biodiesel, thereby reducing the cloud and pour points. Alternatively, the incorporation of cold-flow improver additives can inhibit wax crystal growth and agglomeration, enhancing fuel flowability at lower temperatures. Blending the synthesized biodiesel with petroleum diesel or biodiesel derived from more unsaturated feedstocks, such as soybean or sunflower oil, has also been reported to effectively improve cold-flow properties while maintaining acceptable fuel quality.^77^

The acid value, expressed in mg KOH/g, quantifies the concentration of free fatty acids (FFA) in a fuel sample. Biodiesel standards specify a maximum acid number of 0.5 mg KOH/g. Elevated acid values in biodiesel are undesirable due to their corrosive impact on vehicle fuel systems, underscoring the need for proper storage to prevent oxidative degradation and preserve fuel quality.^78^ The acid value of the *Camelus dromedarius* biodiesel was 0.11 ± 0.05 mg KOH/g, while its FFA content was 0.056 ± 0.02%, both within acceptable limits.

The saponification value of *Camelus dromedarius* biodiesel was 184.66 ± 3.5 mg KOH/g, well below the ASTM D6751 limit of 370 mg KOH/g, and comparable with other animal-based biodiesels as shown in Table 12. This indicates a predominance of medium- and long-chain fatty acids that affect fuel stability and combustion. Because the saponification number is inversely related to molecular weight and carbon chain length, longer carbon chains result in lower values; likewise, increased unsaturation also reduces the saponification number.^79^

The heating value is an essential thermal property, defined as the energy released from biodiesel fuel during complete combustion. The heating value of *Camelus dromedarius* waste biodiesel was determined to be 39.12 ± 2.5 MJ/kg. Currently, no standard limit exists for biodiesel heating value. A higher heating value corresponds to lower fuel consumption, and it is strongly influenced by moisture content. Heating value also tends to rise with increased viscosity and longer carbon chains, while greater degrees of unsaturation (double bonds) reduce it.^79^

The flash point of *Camelus dromedarius* biodiesel was determined to be 154 ± 2 °C, exceeding the standard limits specified by ASTM D6751 (130 °C) and EN 14214 (101 °C). This value is compared with data from the literature presented in Table 12. The flash point, defined as the minimum temperature at which sufficient vapor is produced to ignite in the presence of an ignition source, is strongly influenced by carbon chain length and fatty acid saturation. Biodiesel typically exhibits a higher flash point than conventional diesel, providing enhanced safety during handling and storage.^80,81^

The cetane number and iodine value of *Camelus dromedarius* biodiesel were measured at 68.69 ± 0.57 and 31.84 ± 2.0 g I_2_/100 g, respectively. The cetane number exceeds the EN 14214 and ASTM minimum requirement of 51, ensuring reliable ignition quality, and this aligns with the general observation that saturated methyl esters tend to exhibit higher cetane numbers. The iodine value is well below the EN 14214 maximum limit of 120 g I_2_/100 g, indicating low unsaturation and improved resistance to oxidation. Biodiesels with lower iodine values are usually more combustible and efficient but can show weaker cold-flow performance, as iodine value directly reflects the degree of unsaturation, which influences oxidative stability and fuel behavior.^79,82^ Oxidation stability, which is strongly connected to cetane number, is an important quality parameter that determines the storage durability of biodiesel. The oxidation process is mainly associated with the presence of unsaturated fatty acid methyl esters, particularly polyunsaturated components containing multiple double bonds. During storage, oxidation can result in the formation of peroxides, aldehydes, acids, and polymeric compounds, which negatively affect fuel properties and engine performance. Due to the relatively high saturated fatty acid content of camel fat, biodiesel produced from this feedstock is expected to possess improved oxidation resistance compared with biodiesel derived from highly unsaturated vegetable oils. Nevertheless, storage conditions and exposure to oxygen, heat, and light should be carefully controlled to minimize oxidative deterioration. The addition of suitable antioxidants may also be considered to further enhance storage stability.^83^

Overall, the physicochemical properties of the biodiesel synthesized from *C. dromedarius* fat waste oils are largely consistent with ASTM D6751 and EN 14214 specifications, apart from its cold-flow characteristics. Improvements are recommended to enhance parameters, such as the pour point and cloud point.

Beyond the technical performance of the synthesized biodiesel, the utilization of *C. dromedarius* fat waste offers important sustainability advantages. As an underutilized byproduct of the meat processing industry, camel fat waste represents a low-value residue that can be converted into a renewable transportation fuel, thereby supporting the principles of the circular bioeconomy through waste valorization and resource recovery. The use of this nonedible feedstock also avoids competition with food resources associated with edible vegetable oils, contributing to improved food security and more sustainable biomass utilization. Furthermore, diverting animal fat waste from disposal pathways may reduce the environmental burden associated with waste management while partially substituting for fossil diesel, which has the potential to lower greenhouse gas emissions over the fuel life cycle. Although the present study did not include a quantitative life-cycle assessment or techno-economic analysis, the favorable biodiesel yield and fuel properties obtained suggest that *Camelus dromedarius* fat waste is a promising feedstock for sustainable biodiesel production. Future studies should therefore incorporate comprehensive environmental and economic assessments to quantify the overall sustainability and commercial feasibility of this biofuel production pathway.

## 4 Limitations and Future Work

Even though the current study provides a promising assessment of biodiesel production from *C. dromedarius* fat waste, several limitations should be acknowledged. First, the feedstock was collected from a single source, and variations in camel fat composition from different regions or processing conditions may influence the biodiesel yield and properties. Second, freeze-drying was employed to reduce the moisture content, but this method is energy-consuming and may affect the overall sustainability of large-scale production. Third, the study focused on process optimization and fuel characterization, while broader sustainability evaluations such as life-cycle assessment (LCA) and techno-economic analysis (TEA) were beyond the scope of this work. In addition, engine performance and exhaust emission tests were not conducted, and therefore, the practical applicability of the synthesized biodiesel under real engine operating conditions remains to be verified. Furthermore, although sodium hydroxide was successfully used as the catalyst, its recovery and reusability were not investigated. Future studies should therefore evaluate catalyst recycling to improve the process sustainability and reduce production costs. Additional research should also investigate cold-flow improvement strategies, validate the process using camel fat waste from multiple sources, and perform comprehensive LCA and TEA to assess the environmental and economic viability of large-scale biodiesel production.

## 5 Conclusions

This study demonstrates that *Camelus dromedarius* fat waste is a promising and sustainable nonedible feedstock for biodiesel production, providing an effective approach for valorizing animal waste into renewable energy. Response Surface Methodology successfully optimized the base-catalyzed transesterification process, while FTIR and GC–FID analyses confirmed the successful conversion of camel fat oil into biodiesel. The produced biodiesel generally complied with ASTM D6751 fuel quality requirements, indicating its potential as an alternative diesel fuel, although further improvement of its cold-flow properties is required for low-temperature applications. The scientific contribution of this work lies in establishing the feasibility of utilizing underexplored *Camelus dromedarius* fat waste for biodiesel production through optimized processing conditions and comprehensive fuel characterization. The findings also highlight the potential of camel fat waste for waste-to-energy and circular bioeconomy applications, particularly in camel-producing regions. Future research should focus on improving cold-flow properties, validating the process using different feedstock sources, evaluating engine performance and emissions, and conducting life cycle and techno-economic assessments to support large-scale implementation. The synthesized biodiesel demonstrates suitable physicochemical properties for future engine evaluation.

## Data Availability

Data sets and analyses from this study can be provided by the corresponding author upon reasonable request. All data in this study are presented in the manuscript.
